# A Comparative Study Between Resilon and Gutta-Percha as a Secondary Root Canal Filling Materials: An In Vitro Study

**Published:** 2010-08-15

**Authors:** Payman Mehrvarzfar, Mohammad Ali Saghiri, Kasra Karamifar, Zohre Khalilak, Nahid Maalek

**Affiliations:** 1. Department of Endodontics, Dental School, Azad University of Medical Science, Tehran, Iran.; 2. Instructor, Kamal Asgar Research Center (KARC), MI, USA.; 3. Private Practice, Tehran, Iran.

**Keywords:** Apical Microleakage, Fluid Filtration Model, Resilon, Retreatment

## Abstract

**INTRODUCTION:**

Adequate root canal seal following retreatment is essential for a successful outcome. Resilon/Epiphany (R/E) obturation system has been introduced as a substitute for conventional gutta-percha/sealer method. This in vitro study compared the amount of apical microleakage of R/E with gutta-percha/AH26 (GP/AH26) sealer as secondary root canal filling following retreatment in human teeth.

**MATERIALS AND METHODS:**

Fifty human single-rooted lower premolar teeth were selected. After preparing them with ProTaper rotary NiTi instruments, all the canals were obturated using GP/AH26 sealer. After 10 days, all the samples were retreated using the same rotary NiTi instruments. The samples were divided randomly into two experimental groups A and B (n=20) and positive and negative control groups (n=5). In group A, all canals were obturated using GP/AH26 sealer and in group B all canals were obturated using R/E. After one week incubation in 37˚C with 100% humidity, the amount of apical microleakage was evaluated with fluid filtration model. All the apical microleakage data were analyzed with Mann-Whitney U test.

**RESULTS:**

The mean amounts of apical microleakage were 0.317 ± 0.287 and 0.307 ± 0.281 µL/8min (fluid pressure=30 cm H2O) in experimental group A and B respectively; the difference was not statistically significant (P>0.05).

**CONCLUSION:**

R/E seems to be a good alternative for retreatment as a secondary root canal filling material. However, Resilon/Epiphany obturation system does not completely avert microleakage.

## INTRODUCTION

Although great advances have occurred in endodontics, a proportion of initial root canal treatments (RCT) still fail. Patients’ increased insistence for preserving their natural teeth, as well as advances in non surgical retreatment of the root canals has encouraged endodontists as well as dentists to carry out endodontic retreatments.

Microleakage through the root canal obturating material is a well known cause of RCT failures [1]. The penetration of microorganisms and/or their by-products via micro-gaps in the obturating material can lead to persistent or refractory apical periodontitis [2]. Gutta-percha, a rubber and zinc oxide based material, combined with a sealer are common root canal obturating materials. Resilon (Resilon Research LLC, Madison, CT) is a thermoplastic synthetic (polycaprolactone) polymer based obturating material with bioactive glass and radio opaque fillers. It is biocompatible, non mutagenic, non toxic and also has Food and Drug Administration (FDA) approval [3]. Epiphany (Pentron Clinical Technologies, LLC, Wallingford, CT) is a dual cure resin-based sealer which is used in combination with Resilon. Epiphany primer (Pentron Clinical Technologies, LLC, Wallingford, CT) is a self etching acidic monomer and soluble in water. Some investigations indicate that Resilon/Epiphany (R/E) can bond to root canal walls and produce a monoblock system which can significantly reduce the bacterial microleakage in vitro and in vivo [4][5][6]. Also, an increase in fracture resistance can be seen in roots obturated with R/E [7]. However, a portion of research was unable to demonstrate greater resistance to microleakage between root canals obturated with R/E and the gutta-percha/sealer [8]. Previous studies have not evaluated R/E sealability as a secondary obturating material used in failed RCTs (reRCT) obturated with gutta-percha and sealer. This study compared the apical microleakage of R/E and gutta-percha/AH26 (GP/AH26) sealer as secondary obturating materials in root canal retreatment with the fluid filtration method.

## MATERIALS AND METHODS

In this experimental study, 50 human, single rooted lower premolar teeth with developed root apices were selected. All samples had type I anatomy. The initial apical foramen diameters of the teeth were similar to size 20 or 25 K type ISO files. No root fractures and root resorption could be detected under the stereomicroscope. All the samples were placed in 5.25% sodium hypochlorite overnight for surface disinfection and then brushed under tap water to remove remnant debris. Decoronation was performed and working length was determined 1 mm short of the radiographic apex using an ISO 20 or 25 K type file and periapical radiographs.

ProTaper rotary NiTi instruments (Dentsply, Switzerland) prepared the root canals according to the manufacturer’s instruction with Endo IT (VDW, Germany) motor controller. Root canals were instrumented up to F3 (D0=0.03 mm, Taper: 9%). Irrigation between instrumentation was carried out with 2.6% sodium hypochlorite. Finally, smear layer was removed with 17% EDTA (Smear Clear, Sybron Endo, CA). Distilled water was used as final flush to eliminate additional variables. Root canals were dried with several paper points.

The forty teeth in the experimental groups were obturated using lateral condensation technique with GP/AH26 sealer. Root canalled teeth were kept in 37°C and 100% humidity incubator for 10 days; subsequently retreatments were conducted with chloroform and ProTaper rotary NiTi retreatment instrument in both groups. Instruments included D1, D2 and D3 and then F4 and F5 files. 2.6% Sodium hypochlorite was used as an irrigant between each file. EDTA rinse was used for 1 minute and final flushing was performed with distilled water for 1 minute.

Periapical radiographs were taken to assess adequate Gutta-percha/sealer removal (similar to clinical situation). The experimental samples were divided randomly into two groups (A and B). All randomizations were carried out by generating random digits via Microsoft Excel 2007. In group A (n=20) root canals were obturated as before with GP/AH26 sealer by lateral compaction technique. Gutta-percha cone excess were removed by heat from the canal orifice and packed with plugger. In group B (n=20), root canals were obturated using R/E system.

Initially root canals were dried with several paper points without desiccating the canals. Subsequently, the self-etching primer (Pentron Clinical Technologies) was carried into the root canal with its pipette and any excessive primer was removed with a paper point.

Epiphany sealer was carried into the canals with a NiTi finger spreader (Dentsply Maillefer) and all the canal walls were smeared. A #50 master cone of Resilon was inserted into the canal and "tug back" was checked. Once satisfactory, the rest of the canal was laterally compacted with Resilon accessory cones using NiTi spreaders.

For curing the coronal aspect of Epiphany sealer, a light curing unit (Coltolux, Coltene Whaledent Inc., NJ, USA) was used for 40 seconds. Radiographs were taken to ensure the right working length and adequate homogeneity and quality of secondary root filling material.

Canals in the negative control group (n=5) were prepared with GP/AH 26 sealer and 2 layers of nail polish were applied to the entire outer surface of the root. The positive canals in the negative control group (n=5) were prepared with GP/AH 26 sealer; also two layers of nail polish were applied to the entire outer surface of the root.

**Figure 1:**
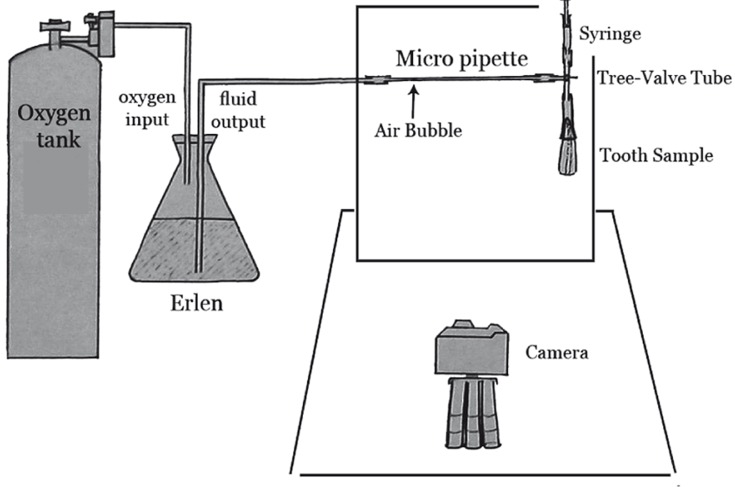
Fluid filtration apparatus

The positive control group (n=5) were obturated with gutta-percha and no sealer. All of the samples were incubated in 37°C and kept in distilled water for 1 week. Fluid filtration model was used to evaluate the amount of microleakage (fluid pressure=30 cm H_2O_) (Figure 1). The interchange of fluid into the micro gaps was determined in a micro liter in eight minutes. Microleakage related to each sample was measured for three times blindly and independently. The mean value was recorded as final apical microleakage value. In this study, all data were analyzed using Mann-Whitney U test. Non-parametric analytic test for comparing the two groups was required; however the data did not follow the normality pattern. Therefore, Kolmogorov-Smirnov test could not be used.

## RESULTS

Data analysis of this study indicated that in R/E group the mean value of microleakage was 0.307±0.281 µL/8min while in GP/AH26 sealer group mean value was 0.317±0.287 µL/8min. This showed no statistically significant difference between the two groups (P=0.11) (Table 1). In the positive control group, microleakage occurred continuously while in the negative no microleakage could be detected.

**Table 1:** Microleakage evaluation results in experimental groups

**Groups**	**Microleakage (µL/8min)**
Resilon/Epiphany	0.307 ± 0.281
Gutta-percha/AH26	0.317 ± 0.287

## DISCUSSION

One of the main success criteria in root canal retreatment is to produce effective seal with secondary root canal fillings. Thus, the microorganisms that were not eradicated and may prevent healing of the periradicular lesions cannot penetrate into the periradicular tissues [6]. Also, an adequate apical seal prevents the penetration of tissue fluids into the canal and therefore impedes re-infection. In the present study, the root canals were initially obturated using GP/sealer and then retreatment was performed. For secondary root canal filling either R/E or GP/sealer was used.

Results showed low mean microleakage values in both groups and no significant differences between groups A and B.

Bodrumlu et al. showed that R/E obturation system produced an adequate apical seal and concluded that this was due to the created monoblock system [9]. Another study demonstrated the superiority of R/E obturant sealing ability when compared with GP/sealer (either AH26 or Seal Apex) using fluid filtration method [10]. A microbial leakage model study also concluded that R/E had greater capability in preventing Streptococcus (S) mutans and Enterococcus (E) Faecalis penetrating into the root canals when compared with GP/AH26 sealer [5]. However, all these studies used R/E as the initial root filling material. Other studies were not able to show a difference in the sealing ability of Resilon/Epiphany system when used as either initial or retreatment root filling material. In all their samples microleakage was present and increased from the first day through to the end of the investigation period (30 days).

In a study with environmental scanning electron microscopy micrographs of reRCT fillings, Resilon tags were shown penetrating into dentinal tubules similar to original Resilon fillings [11]. In our study, after one week, the mean values of microleakage in the secondary root canal fillings with Resilon and GP/AH26 groups were similar. This shows that R/E is just as successful as gutta-percha group in preventing apical microleakage in retreatment cases. The formation of a monoblock obturation system may explain its resistance to microleakage. Adequate root canal shaping in retreatment cases (from F3 through F5) and smear layer removal with EDTA and sodium hypochlorite also contributes to satisfactory debridement and disinfection of the canal. There is a body of evidence that illustrates presence of gaps in R/E root canal fillings challenging the monoblock system formation theory [12][13]. Also, disperse voids were shown in the sealer which may be due to a delay in sealer setting because the Epiphany sealer sets approximately after one week [14]. This delay in setting may lead to separation between Resilon and Epiphany; polymerization shrinkage can also produce stresses which may result in gap formation. Moreover, the complexity of root canal system alone may make the formation of a monoblock system impossible with R/E. This is probably why apical microleakage similar to GP/AH26 sealer occurs. Tay et al. evaluated the sealing quality of the R/E and GP/AH Plus sealer as root canal obturating material in apical region under SEM and TEM and showed that they were similar [8]. We can conclude that currently no method can produce a complete hermetic apical seal in root canals; gaps can still be seen on canal walls [15], similar to our observations. Fluid filtration method was used to determine the apical microleakage. This method was introduced by Derkson et al. and later modified [16][17][18]. This method saves tooth structure, evaluates microleakage three-dimensionally and quantitatively [19]. Also, rotary NiTi instruments have been shown to be more effective in removing GP from root canal system than hand Hedstrom Files [20]; moreover, smear layer was removed to produce better adaptation of secondary root canal fillings with canal walls [21][22].

## CONCLUSION

Resilon/Epiphany can be used as a secondary root canal filling material after root canal retreatment as apical microleakage was minimal with this material. Future investigations should focus on materials that produce a non-permeable seal.
